# The Focal Induction of Reactive Oxygen Species in Rats as a Trigger of Aortic Valve Degeneration

**DOI:** 10.3390/antiox13121570

**Published:** 2024-12-20

**Authors:** Jessica Isabel Selig, Yukiharu Sugimura, Shintaro Katahira, Marco Polidori, Laura Alida Jacobi, Olga Medovoj, Sarah Betke, Mareike Barth, Artur Lichtenberg, Payam Akhyari, Jan-Philipp Minol

**Affiliations:** 1 Department of Cardiac Surgery, University of Dusseldorf, Moorenstrasse 5, 40225 Dusseldorf, Germany; selig@uni-duesseldorf.de (J.I.S.); yukiharu.sugimura@uk-essen.de (Y.S.); shinkatahira@med.tohoku.ac.jp (S.K.); marcopolidori@gmx.de (M.P.); laura.jacobi@mailbox.org (L.A.J.); o.medovoj@ccb.de (O.M.); sarah_betke@web.de (S.B.); mareike.barth@uk-essen.de (M.B.); artur.lichtenberg@med.uni-duesseldorf.de (A.L.); 2 Department of Cardiac Surgery, University of Essen, Hufelandstrasse 55, 45147 Essen, Germany; 3 Division of Cardiovascular Surgery, Tohoku University Graduate School of Medicine, 1-1 Seiryomachi, Aoba-ku, Sendai 980-8574, Japan; 4 Cardiovascular Research Institute Dusseldorf (CARID), University of Dusseldorf, Moorenstrasse 5, 40225 Dusseldorf, Germany

**Keywords:** reactive oxygen species, degenerative aortic valve disease, aortic valve stenosis, photodynamic reaction, mTHCP, temoporfin

## Abstract

Background: Degenerative aortic valve disease (DAVD) is a multifactorial process. We developed an animal model to analyze the isolated, local effect of reactive oxygen species (ROS) on its pathophysiology. Methods: We utilized a photodynamic reaction (PDR) as a source of ROS in the aortic valve by aiming a laser at the aortic valve for 60 min after the administration of a photosensitizer 24 h prior. ROS, laser, and sham groups (*n* = 7 each) for every observation period (t = 0; t = 8 d; t = 84 d; t = 168 d) were established. The amount of ROS generation; morphological changes; inflammatory, immune, and apoptotic reactions; and hemodynamic changes in the aortic valves were assessed using appropriate histological, immunohistological, immunohistochemical, and echocardiographic methods. Results: The ROS group displayed an increased amount of ROS (*p* < 0.01) and increased inflammatory activation of the endothelium (*p* < 0.05) at t = 0. In the ROS group, aortic valves were calcified (*p* < 0.05) and the transvalvular gradient was increased (*p* < 0.01) at t = 168 d. Conclusion: The small animal model employed here may serve as a platform for analyzing ROS’s isolated role in the DAVD context.

## 1. Introduction

Degenerative aortic valve disease (DAVD) is the most common valve-related indicator for surgical or interventional treatment in North America and Europe [1]. The high clinical relevance of DAVD has led to numerous preclinical and clinical studies. Previous research in this field has employed several animal models.

Weiss et al. established a small animal model of low-density lipoprotein receptor-deficient apolipoprotein B-100-only (LDLr^−/−^ ApoB^100/100^) mice fed on normal chow [2]. This model utilizes age and hypercholesterolemia as two of the most important risk factors for atherosclerotic aortic valve degeneration. It was modified using several approaches but always focused on the initial effect of hypercholesterolemia and its consequences on the nexus of degenerative pathways [3,4]. These model variants display a pathophysiological calcification distribution. However, they consider oxidative stress as a consequence rather than as a causative element of DAVD.

Despite its high relevance, little is known about the early features of DAVD. A very common one seen is the extensive remodeling of the cusps with the biomineralization of the lamina fibrosa and transformation of valvular interstitial cells (VICs) and smooth muscle cells (SMCs) into an osteoblast-like phenotype, accompanied by upregulation of the corresponding markers, e.g., osteopontin [5,6,7,8,9].

The very first key factors contributing to this condition are largely unknown. Previous studies have suggested reactive oxygen species (ROS) as key factors in the early cellular and extracellular mechanisms, resulting in the initiation of DAVD [10,11,12,13]. ROS can be found in small animal models already at increased concentrations before any aortic valve dysfunction is apparent. This implies that ROS are initial elements of DAVD [14]. Applied in vitro, ROS promote SMC calcification [10,15]. Furthermore, ROS have been identified as a crucial link in the transduction of pro-osteogenic and pro-fibrotic pathways [16,17]. Moreover, Hajjar et Gotto summarized the relevance of the interaction of oxidative stress and inflammation in cardiovascular degeneration [18].

However, to the best of our knowledge, there is no established animal model employing ROS as isolated and initial causes of DAVD. The development of such a model is necessary to elucidate the role of ROS in this process. Such a model could serve as a platform to evaluate preventive and therapeutic clinical approaches, alongside the well-known axis of hypercholesterolemia and DAVD.

A method to generate ROS directly is through a photochemical or photodynamic reaction (PDR) [19]. Here, the energy of light is transferred to oxygen by means of a biosensitizer. Biosensitizers are chemical substances that are inert and, therefore, have hardly any interactions or effects in the organism [20]. Only through contact with light of an appropriate wavelength is its energy absorbed by conjugated double bonds in these substances before it is transferred to oxygen [21,22].

This oxygen is then dissociated as highly reactive oxygen derivates, mostly ^1^O_2_ and, to some extent, O_2_^−^ and others, summarized as ROS [19]. To avoid any laser-caused tissue alterations, a suitable PDR for in vivo applications should use a low-energy laser, applied to an appropriate biosensitizer [23].

A well-established PDR model used in the cardiovascular field is the combination of rose bengal (tetrachlorotetraiodofluorescein, RB) and a green light laser to cause ROS-mediated reactions in cardiovascular models.

In our previous work, we analyzed the potential of RB and a green light laser to induce ROS for the focal promotion of degenerative changes in the wall of the abdominal aorta in a long-term in vivo model [24]. In addition to the suitability of this model to induce ROS-triggered vascular degeneration, we also investigated the negative aspects of the RB and green light laser model.

The most important negative aspect is the fact that RB has high water solubility. Therefore, it exists at a high concentration in the plasma but not within the target tissue. Another relevant issue is the interaction of the heme group within hemoglobin complexes with the green light laser. Thereby, heme itself acts as a low-potency biosensitizer. Due to this, one cannot distinguish with certainty the source of any potential ROS-mediated effects.

In this context, we seek to focus on the concept of a red light laser (CW; 652 nm, 1 mW, 0.1 W/cm^2^) and the biosensitizer Foscan^®^ (temoporfin, meta-tetrahydroxyphenylchlorin, mTHPC). The latter substance has been particularly well established in the field of oncology, where it has already entered the clinical field as a therapeutic agent. Unlike RB, mTHPC is protein-linked and is thereby absorbable by the blood surrounding the tissue and can thus concentrate there [25]. In an analysis by Kuebler et al., a PDR induced by mTHPC was capable of causing a wide range of local changes in the treated vascular tissue, ranging from slight endothelial hyperplasia up to a thrombus-related total vessel occlusion [26]. However, the principle of this concept involves a ROS-releasing PDR comparable to that achieved by RB and a green light laser. This has already been proven by us to be suitable for the induction of ROS-mediated focal vascular degeneration with atherosclerosis-like features [24]. Our particular adjustment of the PDR in this previous setting, to avoid any acute, laser-caused alterations but to achieve as many ROS as possible, was to use quite a low laser energy (1 mW, 0.1 W/cm^2^), on the one hand, but quite a high concentration of the biosensitizer, on the other hand, with a duration of irradiation of one hour. We transferred the same idea to the setting in the project described here, using a laser of 1 mW, 0.1 W/cm^2^, with a duration of irradiation of one hour and a concentration of mTHCP of 0.3 mg/kg bodyweight, which was associated with the biggest effects by Kübler et al. [26]. The additional advantage of the mTHPC-related PDR is (a) the effect location in the blood surrounding the target tissue, (b) the absorption by the target tissue, and (c) the established concept of its intravascular catheter-guided laser application. Therefore, a PDR employing mTHPC combined with a red light laser may be a very promising approach to inducing ROS-mediated changes in the aortic valve in order to initiate consecutive degeneration and calcific stenosis, which is expected to more closely resemble the features of human calcific aortic valve stenosis.

## 2. Materials and Methods

### 2.1. Animals and Dietary Regimen

Male Wistar rats (250 g) purchased from Janvier Labs, France, were fed ad libitum with standard rat chow enriched with 1.5% dicalcium phosphate and 2% cholesterol beginning one week before the primary intervention. At t = 28 d after the primary intervention, the diet was switched to standard chow to avoid any diet-caused degeneration and calcification.

All surgical procedures and animal experiments were performed in compliance with the *Guide for the Care and Use of Laboratory Animals*, as published by the US National Institutes of Health (NIH Publication 85-23, revised 1996) and were approved by the Committee on the Ethics of Animal Experiments of North Rhine-Westphalia (LANUV), Germany; approval no.: 84-02.04.2017.A153.

All surgeries were performed under isoflurane anesthesia and differentiated analgesia. All efforts were made to minimize suffering.

Induction of ROS: the photosensitizer (Foscan^®^ = temoporfin = meta-tetrahydroxyphenylchlorin = mTHPC by Biolitec, Jena, Germany) was injected in the tail vein at a dosage of 0.3 mg/kg bodyweight under sterile conditions and under general anesthesia. Afterwards, the animals were protected from direct light.

**Figure 1:**
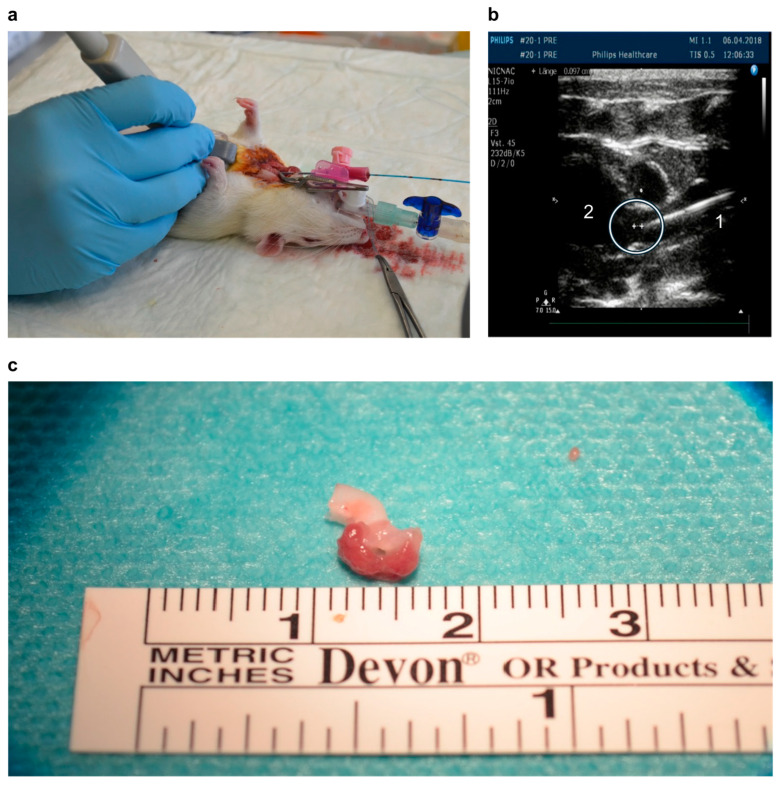
For the ROS, laser, and sham groups, a laser fiber was inserted under echocardiographic control. (**a**) The label “1” in (**b**) shows the fiber; the circle in (**b**) indicates the aortic valve. The label “2” in (**b**) indicates the left ventricle. (**c**) A typical specimen of the aortic valve in the aortic root with the adjacent parts of the myocardium and the aorta.

After 24 h, the PDR was performed under general anesthesia. Before and directly after surgery, the animals received analgesia (buprenorphine, 0.05 mg/kg; Animalcare Limited, York, UK). During the procedure, the animals were ventilated. Fully effective anticoagulation was performed by intravenous application of unfractionated heparin (300 IU/kg). A laser fiber (Biolitec, Jena, Germany) connected to a laser generator (Roithner, Vienna, Austria) was inserted via the right carotid artery to target its tip just above the aortic valve under echocardiographic control (HD11 XE Ultrasound system, 15 MHz, Philips Healthcare, Hamburg, Germany) (Figure 1). At activation, a red light laser (CW, 652 nm, 1 mW, 0.1 W/cm^2^) with a linear emission profile at the tip of the laser fiber was aimed at the aortic valve for 60 min.

For the laser groups, the laser was applied without any prior administration of the biosensitizer.

For the sham groups, the fiber was inserted but neither was the laser activated, nor was any biosensitizer applied beforehand.

Afterwards, the animals recovered from anesthesia and received analgesia as described above. Buprenorphine was administered every 12 h for 2 d after surgery. The animals were visited and clinically assessed at least once a day during the entire follow-up period.

### 2.2. Study Design

ROS, laser, and sham groups of *n* = 7 animals each were established to analyze the effects immediately after irradiation (t = 0) and within a short-term (t = 8 d), mid-term (t = 84 d), and long-term period (t = 168 d).

For the analysis of the direct procedure-induced effects like oxidative stress or apoptosis, immunohistochemical staining for 8-hydroxydeoxyguanosine (8-OHdG, also known as 8-Oxo-2′-deoxyguanosine) and cleaved-caspase-3 were performed at t = 0 and t = 8 d. To characterize potential immunoreactions, we evaluated the animals at each timepoint (t = 0, t = 8 d, t = 84 d, and t = 168 d) for vascular cell adhesion molecule 1 (VCAM-1) expression and for CD 68 to detect monocytes. To assess aortic valve degeneration and hemodynamic changes, von Kossa staining and echocardiography were conducted at t = 0 and t = 168 d.

### 2.3. Explantation and Tissue Analysis

Before the animals were killed by exsanguination under general anesthesia and analgesia, the aortic valve was assessed using echocardiography (Philips Healthcare, Hamburg, Germany with HD11 XE Ultrasound system, 15 MHz) by the same operator as that for the initial procedure at t = 0 and t = 168 d (for t = 168 d, this was performed in a blinded fashion). Afterwards, the aortic root and the proximal part of the aorta were explanted (Figure 1c).

Tissues for histology and immunohistochemistry were embedded in TissueTek^®^ mounting medium (Sakura Finetek, Alphen aan den Rijn, The Netherlands) at −20 °C and were cut into 5 μm cryosections using a CM 1950 cryostat (Leica Biosystems, Wetzlar, Germany) in order to achieve at least one tissue section per specimen and assay with all three cusps of the aortic valve. Histological staining including von Kossa staining was performed as described elsewhere [27].

Immunohistochemical staining was performed according to protocols that we have described previously [27]. We applied primary antibodies for 8-hydroxydeoxyguanosine (8-OHdG; JaICA, Chiyoda, Japan), cleaved-caspase-3 (Cell Signaling Technology, Danvers, MA, USA), CD 68 (Abcam, Cambridge, UK), vascular cell adhesion molecule 1 (VACM-1; Abcam, Cambridge, UK), vimentin (Progen, Heidelberg, Germany), and von Willebrand factor (vWF; Dako Cytomation, Glostrup, Denmark). Secondary antibodies were conjugated to diaminobenzidine (DAB; Zytomed Systems, Berlin, Germany) or the fluorophores Alexa 546 and 488 (Invitrogen, Carlsbad, CA, USA). For immunohistochemical staining targeting cleaved-caspase-3, hemalum was applied as a counterstain (Merck KGaA, Darmstadt, Germany). For fluorescence staining, the sections were covered with mounting medium containing 4′,6-diamidino-2-phenylindole (DAPI; Carl Roth, Karlsruhe, Germany). Image acquisition and processing were performed as described above.

For imaging, we used a DM2000 microscope system with a DFC 425C digital camera (Leica Microsystems, Wetzlar, Germany) and Leica Application Suite V3.7 software, supported by a fluorescent lamp (EL6000, Leica Microsystems, Wetzlar, Germany) whenever necessary. Microscope objectives were used according to the requirements: 400× magnification for 8-OHdG, CD68, and von Willebrand factor (vWF); 100× magnification for cleaved-caspase-3, VCAM-1, and von Kossa.

For image processing and quantification, we used the program ImageJ 1.52n (U. S. National Institutes of Health, Bethesda, MD, USA) supported by The BioVoxxel Image Processing and Analysis Toolbox (Brocher, Hermance, Switzerland, 2015, EuBIAS-Conference, 2015).

### 2.4. Evaluation of 8-OHdG

Oxidative stress was detected indirectly by measuring 8-OHdG, an oxidized derivative of guanosine found in DNA and RNA formed by the presence of ROS.

For the quantitative evaluation, images of the DAPI staining with a wavelength range of 340–380 nm and the 8-OHdG staining with a wavelength range of 515–560 nm were taken in grayscale. A total of nine representative regions per aortic valve were photographed.

The 8-OHdG-specific signal in the cell nucleus of the valve tissue was measured, as this is representative of the effect of oxidative stress on the DNA of the cells. For this purpose, the DAPI images were first processed with ImageJ 1.52n using a specially created macro adapted to the images so that the cell nucleus outlines could be extracted and transferred to the 8-OHdG images as the region of interest. The average grayscale value of the 8-OHdG signal was then measured specifically in the cell nuclei.

These measurements were carried out with all images of a valve, resulting in nine individual values for each specimen. The mean values of each aortic valve were normalized to the results of the positive control (aortic valve tissue with previous treatment with 3% H_2_O_2_ solution for 30 min) to exclude differences in the intensities of the individual staining runs.

The grayscale images were finally colored using ImageJ 1.52n to make the results clearer. The DAPI signals are shown in blue and the 8-OHdG signals in red.

### 2.5. Evaluation of VCAM-1

For the semiquantitative evaluation of theVACM-1 staining, we established a scoring system (0–5) adapted to the von Kossa staining score described in detail before [28,29,30]. Here, 0 equals no staining at all, 1 equals single focal areas of staining, 2 equals several broader areas of staining, 3 equals less than 50% staining, 4 equals more than 50% staining, and 5 equals total staining. For each aortic valve, defined areas of the annulus and the cusps (in total nine images per specimen) were evaluated in a blinded fashion as described, and their arithmetic means were calculated.

### 2.6. Evaluation of CD 68

During the quantitative evaluation of CD 68, DAPI images were used to manually mark the tissue boundaries as the region of interest, which afterwards were transferred to CD 68 images. The proportion of CD 68-specific signal within the region of interest was then calculated with the help of a self-created macro in ImageJ 1.52n. The area fraction of CD 68-positive signal is given in % of the total tissue area and allows comparable analyses between the different specimens. A total of nine representative regions per aortic valve were photographed.

### 2.7. Evaluation of Von Kossa Staining

For the semiquantitative evaluation of the von Kossa staining, we performed the von Kossa staining score (0–5) as previously described [28,29,30]. Here, 0 equals no calcification at all, 1 equals slight brownish staining suggesting the beginning of calcification, 2 equals areas of distinctive calcification, 3 equals extended focal areas of calcification but less than 50%, 4 equals 50–75% calcification, and 5 equals > 75% calcification of the analyzed area. For each aortic valve, defined areas of the annulus and the cusps were evaluated in a blinded fashion as described. The arithmetic means were calculated for the annulus and the cusps separately.

### 2.8. Statistical Analysis

The number of animals per group was determined by a power-based sample size calculation (G*Power 3) [31]. The effect size estimation was adapted from Roosens et al. [32], as well as our own preliminary research experience. We chose the *t*-test for independent samples as the underlying statistical test. Given a significance level (alpha) not exceeding 5% and a power (1-beta) of at least 80%, we estimated a sample size of *n* = 7 animals per research arm.

Values are presented as scatter dot plots, representing the means ± standard error of the mean (SEMs; Prism7, GraphPad Software, La Jolla, CA, USA). Inter-group comparisons were performed using a one-way ANOVA, followed by a parametric Tukey’s post hoc test or the nonparametric Dunn’s post hoc test according to the requirements (Prism 7, GraphPad Software, La Jolla, CA, USA). Probability values less than 0.05 were considered statistically significant.

## 3. Results

**Figure 2:**
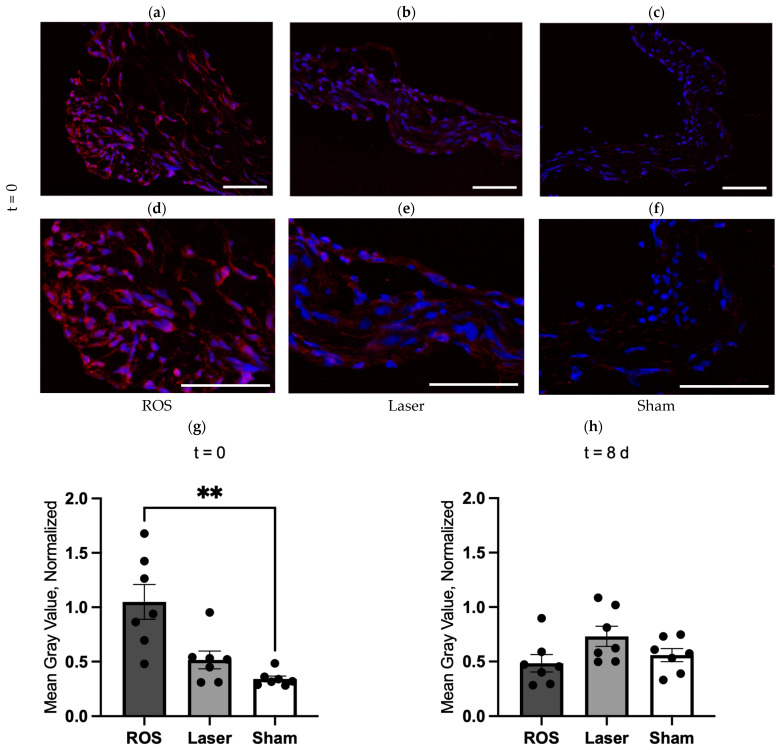
Indirect ROS detection by 8-OHdG fluorescence. After the initial procedure, the content of generated ROS was assessed indirectly via 8-OHdG fluorescence at t = 0 and t = 8 d ((**a**,**d**) = ROS; (**b**,**e**) = laser; (**c**,**f**) = sham at t = 0). Quantitative analysis of 8-OHdG (mean gray value normalized to the positive control of the staining run) displayed an increase in the laser and ROS groups at t = 0, with a significant difference between the ROS and the sham group (**g**). On the contrary, no differences between the groups were found at t = 8 d (**h**). 8-OHdG (red) with DAPI counterstain (blue); scale bars = 50 µm. ** *p* < 0.01.

The specimens at t = 0 d and t = 8 d were stained for 8-OHdG as an indirect marker of increased oxidative stress (Figure 2). The distinctive appearance of the cusps compared to the annulus was observed. The quantitative analyses of the 8-OHdG fluorescence at t = 0 displayed an increase in the laser and ROS groups, with the maximum values in the latter. Moreover, we found a significant difference (*p* < 0.01) between the ROS and sham groups. On the other hand, at t = 8 d, no differences between the sham, laser, and ROS groups could be observed.

**Figure 3:**
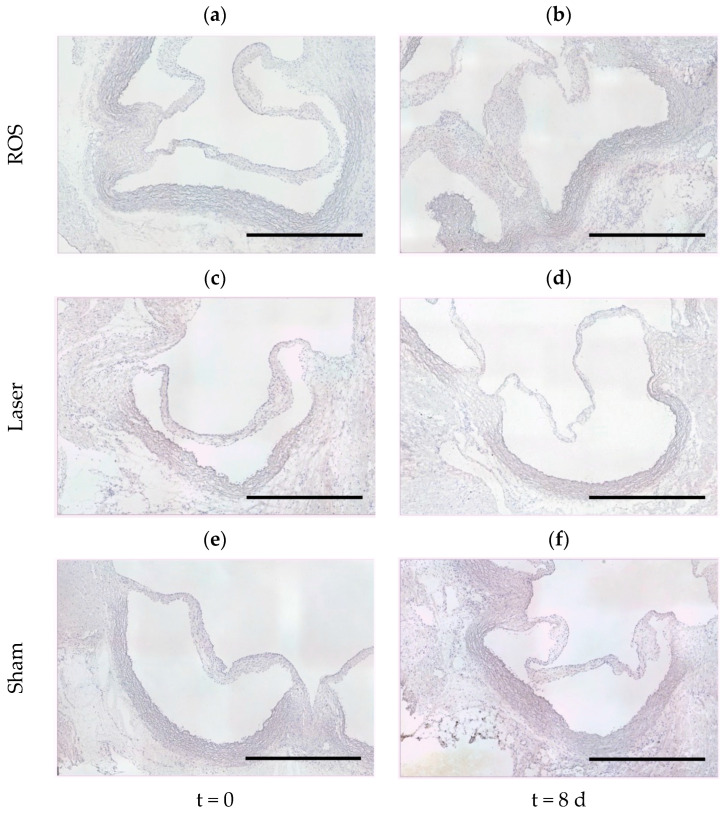
Analyses of the cleaved-caspase-3 activity. Neither the cross-section images of the ROS groups at t = 0 (**a**) and t = 8 d (**b**) nor those of the corresponding laser (**c**,**d**) and sham groups (**e**,**f**) displayed distinctive cleaved-caspase-3 activity. Immunohistochemistry: cleaved-caspase-3 (DAB, brown) with hemalum counterstain (blue); scale bars = 1000 µm.

In parallel to the analysis of 8-OHdG fluorescence, cleaved-caspase-3 immunohistochemical staining was used to detect signs of apoptosis at t = 0 and t = 8 d. No enhanced activity was detected in any group (Figure 3).

**Figure 4:**
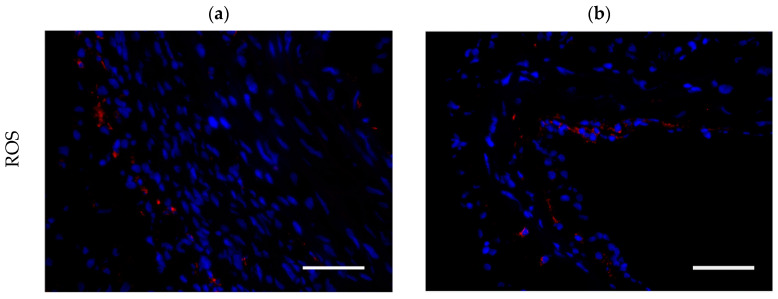

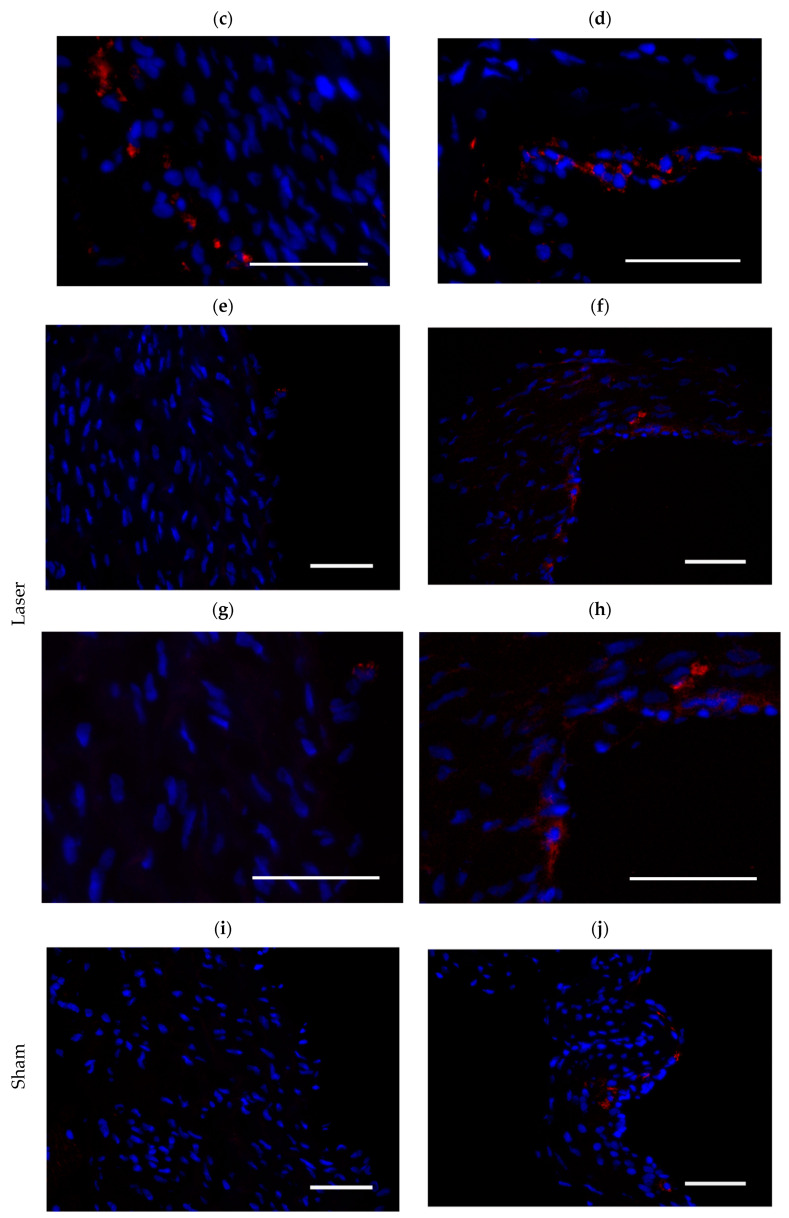

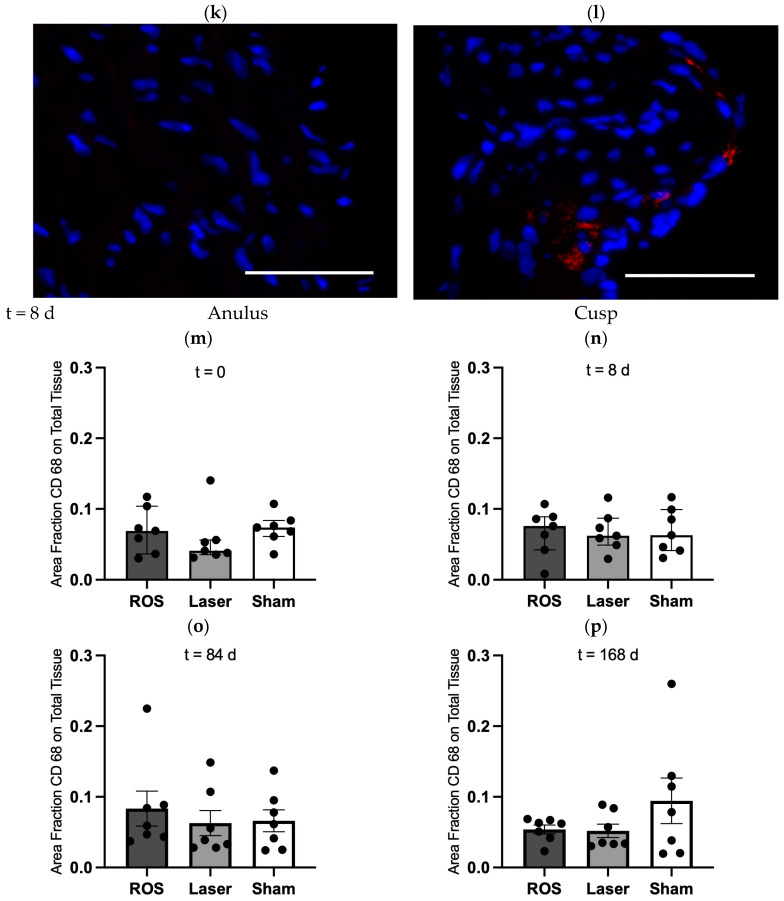
Concentration of monocytes was quantitatively assessed via CD 68 expression. For this purpose, the percentage of CD 68-specific signals in the total tissue area was calculated. Representative cross-section images at t = 8 d (ROS group: (**a**,**c**) = annulus, (**b**,**d**) = cusp; laser group: (**e**,**g**) = annulus; (**f**,**h**) = cusp; sham group: (**i**,**k**) = annulus; (**j**,**l**) = cusp). Quantitative analysis (**m**–**p**). CD 68 (red) with DAPI counterstain (blue); scale bars = 50 µm.

In order to analyze the level of inflammation quantitatively, we assessed the concentration of CD 68-positive cells (monocytes; Figure 4). There was a broad distribution of these values, especially in the groups at t = 84 d and t = 168 d; no relevant differences or tendencies were observed.

**Figure 5:**
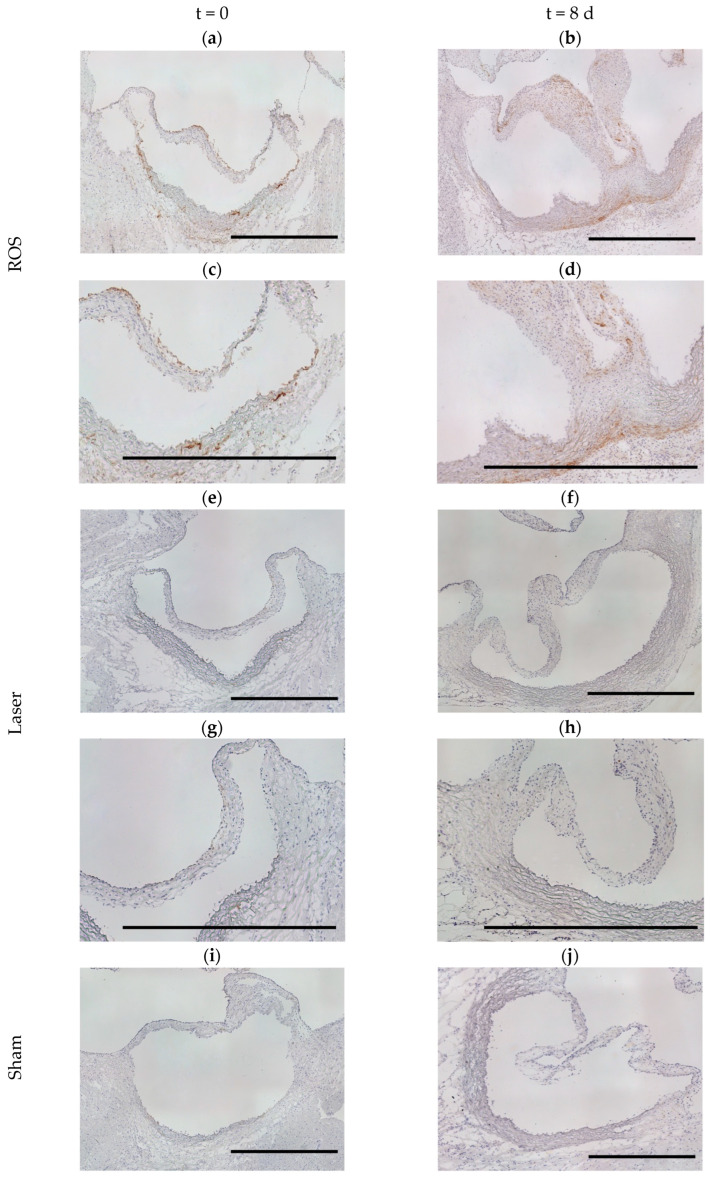

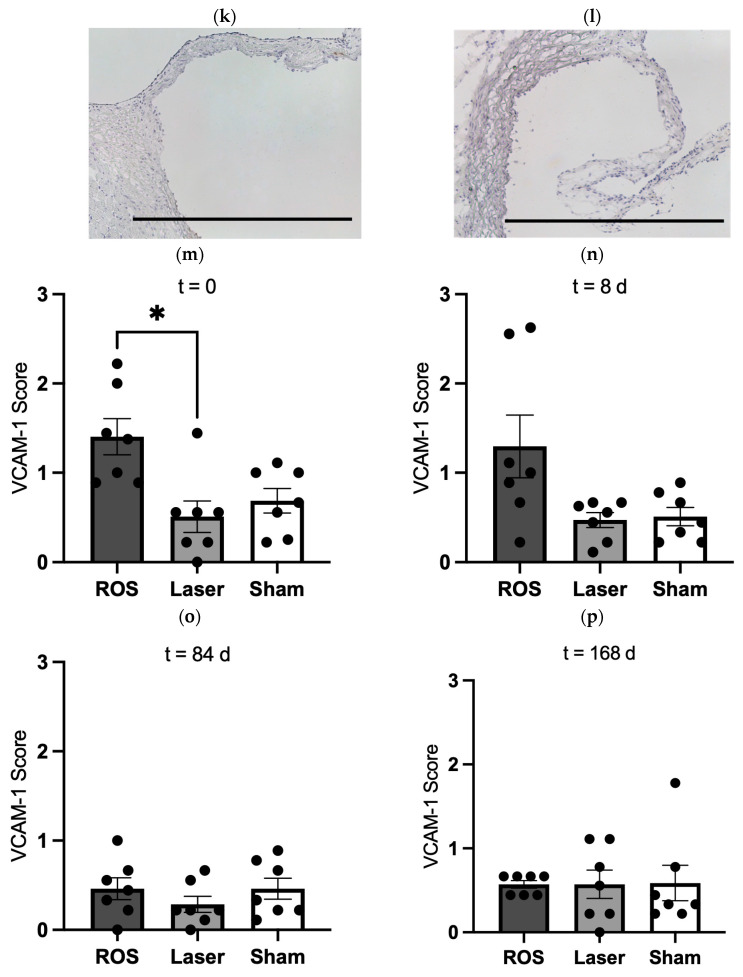
Analysis of the VCAM-1 expression. Representative cross-section images of the ROS groups at t = 0 (**a**,**c**) and at t = 8 d (**b**,**d**) displayed the increased expression of VCAM-1 compared to their respective control groups (laser group at t = 0 (**e**,**g**) and t = 8 d (**f**,**h**); sham group at t = 0 (**i**,**k**) and t = 8 d (**j**,**l**)). Semiquantitative analysis of VCAM-1 expression ((**m**–**p**); 0 = no expression; 5 = maximum). Immunohistological staining of VCAM-1 (DAB, brown) with hemalum counterstain (blue); scale bars = 1000 µm. * *p* < 0.05.

The endothelial inflammatory response was assessed in terms of VCAM-1 expression. The ROS groups at t = 0 and t = 8 d displayed an increased expression of VCAM-1 compared to the corresponding laser and sham groups. This resulted in a significant difference (*p* < 0.05) between the ROS and laser group at t = 0. Nevertheless, this distinctive difference was not observed at t = 84 d or at t = 168 d (Figure 5).

**Figure 6:**
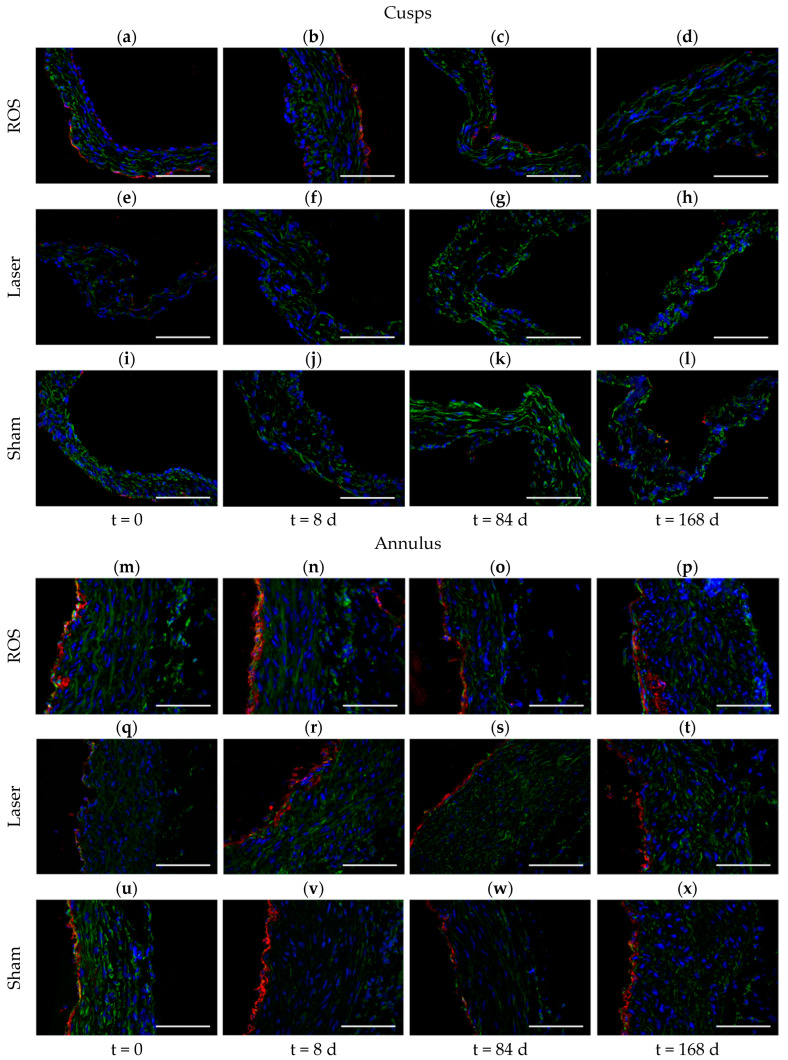
Analysis of the endothelial layer. While the cusps (**a**–**l**) displayed hardly any remaining endothelial layer (red = vWF) at any timepoint, neither in the ROS (**a**–**d**) nor in the laser (**e**–**h**) or the sham groups (**i**–**l**), the annulus area displayed a continuous, lumen-sided endothelial layer regardless of the timepoint or group (**m**–**x**). vWF (red), vimentin (green), with DAPI counterstain (blue); scale bars = 100 µm.

The immunohistochemical staining for vWF revealed the scarce existence of a discontinuous endothelial layer at the cusps at each timepoint (t = 0; t = 8 d; t = 84 d and 168 d) and for each group (ROS, laser, and sham groups; Figure 6a–l). Contrary to this finding, the annulus area displayed a continuous, lumen-sided endothelial layer regardless of the timepoint and the group (Figure 6m–x).

**Figure 7:**
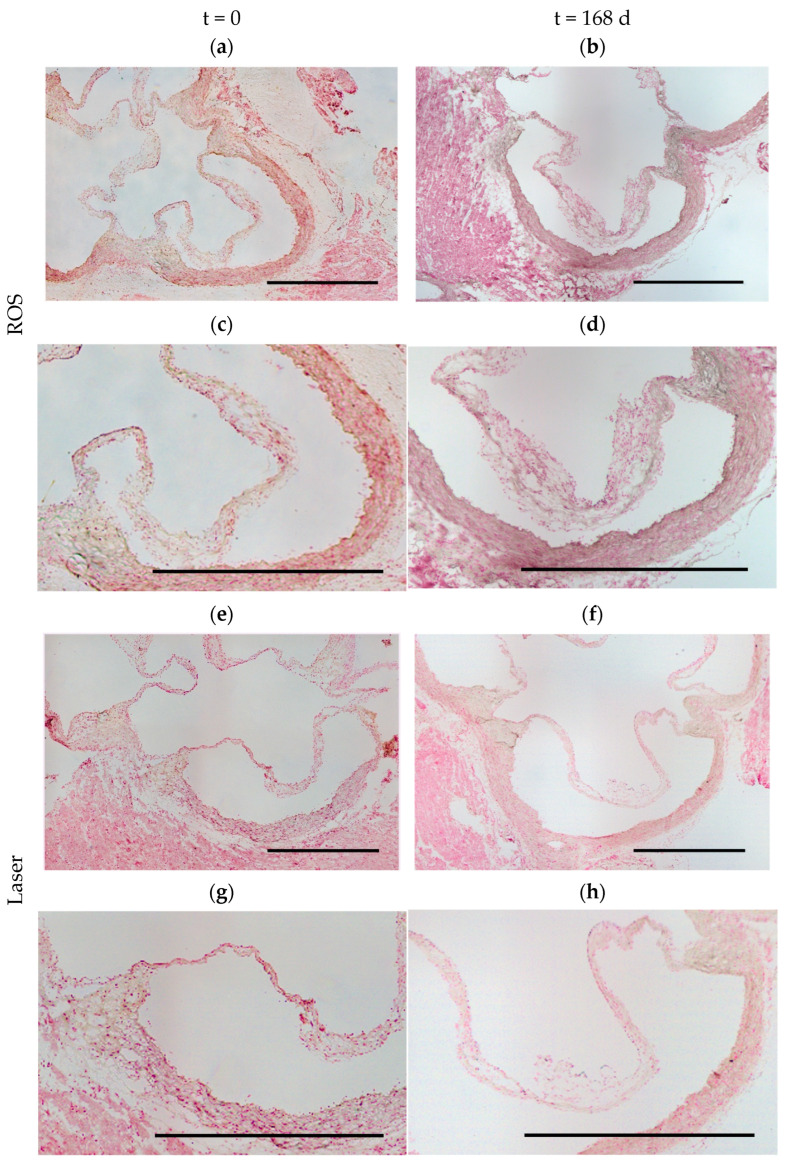

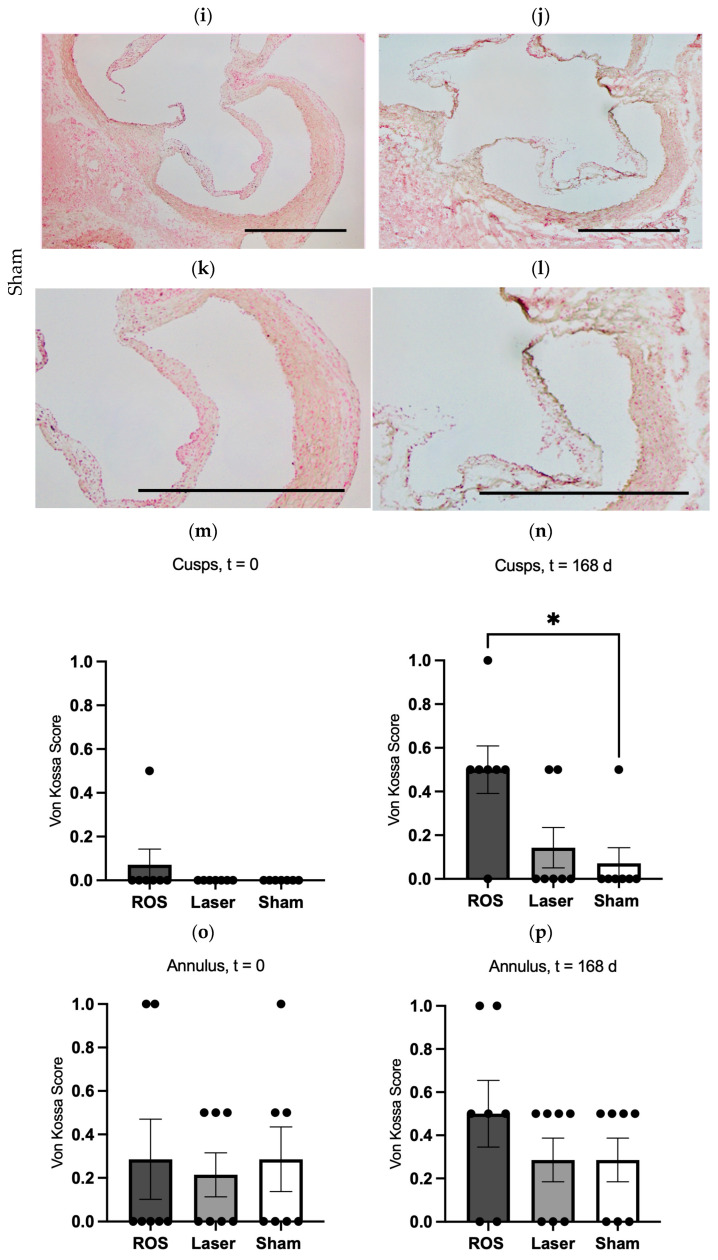
Analysis of degenerative calcification. Representative pairs of cross-section images and their details for all groups at t = 0 (ROS (**a**,**c**), laser (**e**,**g**), sham (**i**,**k**)) and at t = 168 d ((ROS (**b**,**d**), laser (**f**,**h**)**,** sham (**j**,**l**)). Von Kossa staining: calcification (brownish/black) measured with nuclear fast red counterstain (red); scale bars = 1000 µm. This analysis showed hardly any hint of calcification in the cusps at t = 0 (**m**) but a slight increase over time, especially in the ROS group, which achieved significant differences at t = 168 d (**n**). * *p* < 0.05. On the contrary, the results of the semiquantitative analysis (von Kossa score: 0–5) displayed slight hints of calcification in the annulus areas in some of the animals at t = 0 and t = 168 d, regardless of the groups, with hardly any aggravation over time (**o**,**p**).

The degenerative calcification was assessed semiquantitatively by means of the von Kossa score (0–5) in a blinded fashion in the area of the cusps and the annulus separately at t = 0 and t = 168 d (Figure 7). This analysis displayed slight hints of calcification in the annulus areas in some of the animals at t = 0, regardless of the groups, with hardly any aggravation over time. On the other hand, this analysis showed hardly any hint of calcification in the cusps in any of the groups at t = 0 but an increase over time, especially in the ROS group. After 168 days, there was an increased von Kossa staining indicative of slight calcification of the cusps in the ROS group, which was significantly higher than in the sham group at 168 days, and also when compared to the initial values of the ROS group (at t = 0).

**Figure 8:**
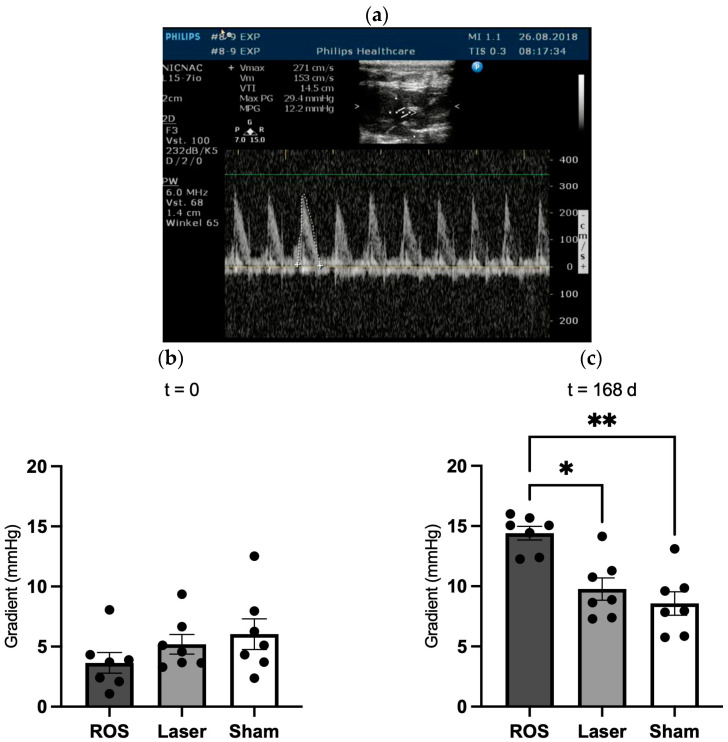
Analysis of the mean transvalvular pressure gradient. The measurement of the mean transvalvular pressure gradient just before termination of each experiment (**a**) displayed no significant differences at t = 0 (**b**), but an increase in all the groups over time up to t = 168 d (**c**) with a significant difference between the ROS and laser group as well as the ROS and sham group. * *p* < 0.05; ** *p* < 0.01.

The measurement of the mean transvalvular pressure gradient just before the termination of each experiment at t = 0 and t = 168 d displayed no significant difference at t = 0. At t = 168 d, a distinctive increase in all the groups could be observed, which achieved significance between the ROS and the laser group (*p* < 0.05) and the ROS and the sham group (*p* < 0.01) (Figure 8).

## 4. Discussion

In this study, we established an animal model analyzing the potential of a PDR between mTHPC and red laser energy as a mechanism leading to ROS-mediated aortic valve degeneration. The successful generation of a significant amount of ROS was proven. Any direct degenerative effects, such as apoptosis and necrosis, were excluded. After an initial endothelial inflammatory reaction, the ROS group displayed a slight but significant calcification in the aortic valve cusp area that was in parallel to a significantly increased transvalvular gradient.

In previous models that employed a PDR as a source of ROS in the vascular system, immediate thrombus generation in that area was observed occasionally [24]. This is thought to be because of impaired endothelial function or even damage [33,34] or as a result of the activation of circulating platelets [35,36]. We chose a light source that was much less intense (0.1 W/cm^2^) compared to the one employed, e.g., by Kikuchi et al. (0.9 W/cm^2^), as well as in the lower area of the range of photochemical laser effects and very distinctive below the ranges of thermal- or photoablative laser effects according to Boulnois [23,34].

Our analysis of the endothelium did not provide any clues of impaired function or structure. At each timepoint, the sham, laser, and ROS groups displayed an intact endothelial layer along the surface of the aortic valves in the annulus area, but also a broken line of vWF expression in the cusp areas.

We do not consider this as definitive proof of the endothelial integrity, but rather a probable processing-caused artifact in the highly sensitive aortic valve tissue, which is especially true for the cusp areas.

However, throughout our experiments, we did not see, macro- or microscopically, any thrombus formation at any time, which was primarily a result of fully effective anticoagulation due to the administration of unfractionated heparin just before the insertion of the laser fiber. We considered this to be mandatory, because a significant thrombus formation in the area of the aortic valve could have harmed the animal seriously. Moreover, we were primarily aiming at the late chronic effects of ROS.

The generated ROS content was immediately analyzed by the assessment of 8-OHdG within the aortic valves. The ROS group displayed a significant increase at t = 0. Moreover, the very wide range of results within the ROS group at t = 0 was remarkable. In addition to other contributing mechanisms, which are discussed later, one must consider the limitations of any analysis of ROS. They are inherently transient and can only be assessed indirectly through their effects. The measurement of 8-OHdG within DNA is a fairly common approach, yet Hamilton et al. reported a half-life of 8-OHdG in the liver of 11 min after exposing mice to ionizing radiation [37]. Hence, even slight differences in the timing of specimen processing, which are not always preventable, directly lead to different results. The use of larger groups of animals in future experiments on this topic could compensate for this artificial effect.

Despite all of these limitations, the obtained results provide a proof of concept for the exposure of the aortic valves to a significant amount of ROS by a PDR.

Our results showed, on the one hand, no difference in the very low level of CD 68-positive cells (monocytes), regardless of the timepoint and groups, but a remarkable increase in the VCAM-1 expression in the ROS group in the first eight days. At first glance, this might sound contradictory. Greenberg et al. summarized the initiation of the calcific aortic valve disease as follows: As well as other risk factors, such as aging, obesity, and hypertension, systemic inflammation triggers damage to the endothelial layer of the aortic valves, allowing for the infiltration and oxidation of lipoproteins, like low-density lipoprotein (LDL) and lipoprotein (a) (Lp(a)) [17,38,39,40,41,42,43,44]. This leads to a chronic inflammatory response, also resulting in an increased expression of endothelial vascular adhesion molecules, including VCAM-1 [45]. If one considers the concentration of CD 68-positive cells as an indicator of systemic inflammation and the VCAM-1 expression as a sign of an endothelial inflammatory response, one could argue that our model accurately represents these important aspects (and most likely the downstream steps) of the known pathways of DAVD without the necessity of inducing chronic inflammation or any of the aforementioned risk factors.

Von Kossa staining showed only a slight calcification tendency in the form of gray tones, but no pattern of advanced aortic valve degeneration as a known endpoint in other publications. Nevertheless, we noted that after 168 days, the cusps of the ROS group repeatedly showed a greater tendency to calcification within the mentioned limits than in the comparison groups, resulting in a significant difference from the sham group. To adequately represent this, we had to perform a separate analysis of cusps and annulus. We interpret this as evidence of an early stage of aortic valve degeneration. To investigate this, further experiments with longer follow-up periods should be considered.

A prevailing cause of ectopic calcification is apoptosis. Here, one has to discuss ROS as activators of elements of the apoptotic pathway, e.g., caspases, when they are present at very high concentrations [46]. This mechanism is inconsistent with our hypothesis that ROS act as triggers of multiple and extensive pathways in the pathophysiology of generative aortic valve disease. Therefore, we consider it as crucial to have excluded this quite direct side effect by showing no detectable caspase activity soon after the PDR. Moreover, the DAPI counterstains did not reveal a reduced number of cells in the areas of calcification, which excludes initial cell death mechanisms like apoptosis and also necrosis as the prevailing reason for the observed calcification.

Byon et al. have described oxidative stress as a trigger of key osteochondrogenic factors that promote the phenotypic switch of SMCs to an osteochondrogenic phenotype [15]. Although SMCs could be detected as being constantly present in the aortic valves [47], their part in the valve composition is small compared to that of VICs. Nevertheless, Greenberg et al. also summarized the mechanisms of ROS-induced osteochondrogenic differentiation of VICs as a source of calcification [38]. After excluding apoptosis and necrosis as sources of the observed calcification, we see our findings as being in line with the effects described in that study [38].

Although no animal displayed at any time any clinical aspects of aortic valve stenosis, we found that the calcification was accompanied by a significant increase in the transvalvular gradient, suggesting changes in the hemodynamic flow profile. We have taken utmost care to avoid methodological bias, e.g., all measurements were performed throughout the study by the same operator. Moreover, we preferred the calculation of the mean gradient compared to the less accurate calculation of the orifice area as recommended by Baumgartner et al. [48]. A paired measurement and analysis of the same animals at t = 0 and t = 168 d would have been an ideal approach from a scientific point of view. However, according to our experience, the tolerance of the animals is quite exhausted after 60 min under anesthesia. To avoid any dropouts caused by further prolongation of anesthesia for a more detailed examination, we chose the approach described above.

Most of our data that were assessed quantitatively or semiquantitatively offered quite a wide range that, together with the small number of animals per group, contributed to the few differences that could be described as statistically significant.

Previous studies have described a certain spread of the mTHPC uptake in the target tissue [49]. Therefore, we consider the finding of Cramers et al. as supportive of this model. This finding showed that mTHPC achieves its peak drug concentration in the heart within the first 1–3 h after injection via the tail vein, with a constant level up to 72 h after injection. At 24 h after injection, the declining mTHPC concentration in the plasma equals the concentration in the heart [25].

The next issue that might contribute to the spread of the results is the positioning of the laser fiber. Although it was placed as close to the aortic valve as possible according to echocardiographic control, a certain level of variation had to be accepted due to the setting itself and due to the constant movement of the heart. This would have had a greater impact in our previous PDR model employing rose bengal as a biosensitizer with a corresponding green light laser [24]. As the heme group in hemoglobin absorbs green light but reflects red light, the blood acts as a kind of insulator of the aortic valve for green light but not for red light. That was a major issue contributing to why we chose this mode of PDR utilizing a red light laser and mTHPC for the intravascular application in this study. In a certain way, the abovementioned aspects have made it possible for the laser from a fiber of one square millimeter to provide a PDR, which covers the entire circumference of the aortic valve in an almost homogeneous manner. With every heartbeat, the angle of the fiber to the aortic valve changes, thereby increasing the irradiation area. In addition, the red laser does not directly hit the aortic valve but is ubiquitously reflected by the heme beforehand, as described above. In addition to the PDR in the tissue, the biosensitizer in the plasma leads to generation of ROS in the blood phase around the aortic valve, which are swirled by the heart and valve actions.

Among these considerations, one also must discuss the general limitations of this model. Exposing the aortic valve to ROS generated by a PDR for 60 min provides just a basic image of the clinical pathophysiology of DAVD in humans. Whereas, in our judgment, prolonging the procedure for longer than 60 min exceeds the tolerance of the animals, it could be proposed that repetitive procedures create a more accurate image of lifelong ROS exposure. Moreover, we used one of the most widespread animal models (male Wistar rats, 250 g), which is available in the short term and allows a certain degree of flexibility during experiments. However, these animals were quite young adult rats, which usually display a higher tolerance towards exogenic effects than older animals, especially regarding long-lasting degenerative processes. We tried to compensate for this by enriching the animals’ diets with 1.5% dicalcium phosphate and 2% cholesterol one week before and four weeks after the PDR. Except for vitamin D, which we did not use here, this is the same as the diet we used previously and for which we analyzed the impact on the serum biochemistry [24]. Our previous analyses and those of the sham groups in this study excluded any diet-caused degenerative effects.

In all of these attempts to compensate for the fundamental limitations of the model, especially the short irradiation time, it must be accepted that the experimental model presented here is not suitable for realistically depicting the chronic and multifactorial character of DAVD. The main purpose of our work, however, was to present the contribution of ROS to this process in as isolated a way as possible.

We think to have achieved this by finding the results of this work according to those in our previous one [24]. There, our much closer analysis at 0, 2, 4, 8, 28, and 56 days revealed, after the dissolving of an initial PDR-caused thrombus formation in the aorta at t = 8 d, no significant morphological difference in an intermediate phase between t = 8 d and t = 56 d. Nevertheless, we found a significantly increased activation of the matrix metalloproteinase (MMP) in the vascular tissue at t = 8 d, followed by a reduced expression of alpha- smooth muscle actin, significant wall thickening, and calcification at t = 56 d. We saw this in line with other authors, who described ROS as promoters of a switch of SMCs to an oteochondrogenic phenotype, thereby migrating and secreting matrix components [15,50]. This gave us the suggestion that this model could be capable of launching ROS-mediated pathways resulting in distinctively delayed morphological changes.

We transferred this established concept with the same parameters but necessary adjustments to target the aortic valve. The crucial link in this work described here between initial ROS exposure by PDR and slight morphological alterations, e.g., calcification and hemodynamic changes is, to our judgement, the significantly increased expression of VCAM-1 in the ROS groups at t = 0 and t = 8 d. As described above, endothelial expression of VCAM-1 is considered a link between common risk factors like aging, obesity, hypertension, and systemic inflammation and calcific aortic valve disease [38,45]. By the absence of these risk factors, including systemic inflammation, one could argue that the VCAM-1 expression in the ROS groups could be seen as an early consequence of the PDR-generated ROS, with mediation of downstream pathways also resulting in morphological changes like slight calcification and hemodynamic alterations of the aortic valve at t = 168 d. This would be in line with the descriptions of the interaction of inflammation and oxidative stress, especially regarding the upregulation of VCAM-1 in the context of cardiovascular degeneration, by Hajjar et Gotto [18].

As discussed above, the model of a PDR of 60 min with the longest follow-up period of 168 d has its limitations in mimicking long-lasting processes, inducing chronic diseases. Nevertheless, we think our model is suitable to provide a contribution to the discussion on the multiple impacts of ROS within this nexus.

## 5. Conclusions

We established an animal model to analyze the isolated and focal effects of oxidative stress in the context of DAVD. We consider the findings of an endothelial inflammatory response and the calcification of the aortic valve accompanied by hemodynamic effects, along with the exclusion of direct short-term effects like necrosis and apoptosis, as clues to the initiation of the currently discussed pathways of DAVD and their endpoints.

After this achievement, the abovementioned drawbacks should be addressed, e.g., consideration of the usage of older animals and the repetitive, if not longer, ROS exposure caused by the PDR.

Nevertheless, this animal model could serve as a platform to analyze the isolated role of ROS in more detail or in combination with other factors in the context of DAVD.

## Data Availability

The original contributions presented in the study are included in the article; further inquiries can be directed to the corresponding authors.
